# Contrasting effects of soil microbial interactions on growth–defence relationships between early‐ and mid‐successional plant communities

**DOI:** 10.1111/nph.17609

**Published:** 2021-08-19

**Authors:** Stefan Geisen, Robin Heinen, Elena Andreou, Teun van Lent, Freddy C. ten Hooven, Madhav P. Thakur

**Affiliations:** ^1^ Department of Terrestrial Ecology Netherlands Institute of Ecology (NIOO‐KNAW) Wageningen 6708PB the Netherlands; ^2^ Laboratory of Nematology Wageningen University Wageningen 6708PB the Netherlands; ^3^ Lehrstuhl für Terrestrische Ökologie, Wissenschaftszentrum Weihenstephan für Ernährung, Landnutzung und Umwelt Technische Universität München Freising 85354 Germany; ^4^ Institute of Ecology and Evolution University of Bern Bern 3012 Switzerland

**Keywords:** bacteria, fungi, growth–defence relationships, plant successions, plant–soil interactions, protists, soil microbiome

## Abstract

- Plants allocate resources to processes related to growth and enemy defence. Simultaneously, they interact with complex soil microbiomes that also affect plant performance. While the influence of individual microbial groups on single plants is increasingly studied, effects of microbial interactions on growth, defence and growth–defence relationships remain unknown, especially at the plant community level.
- We investigated how three microbial groups (bacteria, fungi, protists), alone and in full‐factorial combinations, affect plant performance and potential growth–defence relationships by measuring phenolics composition in early‐ and mid‐successional grass and forb communities in a glasshouse experiment.
- Microbial groups did not affect plant growth and only fungi increased defence compounds in early‐ and mid‐successional forbs, while grasses were not affected. Shoot biomass–defence relationships were negatively correlated in most microbial treatments in early‐successional forbs, but positively in several microbial treatments in mid‐successional forbs. The growth–defence relationship was generally negative in early‐successional but not in mid‐successional grasses. The presence of different microbiomes commonly removed the observed growth–defence relationships.
- We conclude that soil microorganisms and their interactions can shift growth–defence relationships differentially for plant functional groups and the relationships vary between successional stages. Microbial interaction‐induced growth–defence shifts might therefore underlie distinct plant strategies and fitness.

## Abstract 2

See also the Commentary on this article by Bender, **233**: 1015–1017.

## Introduction

Plant species differ profoundly in how much energy they allocate to their vegetative and reproductive growth compared with their defence against natural enemies (Coley *et al*., 1985; Coley, 1988; Herms & Mattson, 1992). In addition, plant growth and abundance are often driven by nutrient availability and the balance between their antagonists and mutualists in the environment (Huot *et al*., 2014; Smakowska *et al*., 2016). As a result, the variation in plant adaptations that are depending on nutrient availability and the biotic environment determines the composition and dynamics of plant communities (Olff & Ritchie, 1998; HilleRisLambers *et al*., 2012). A dramatic example of vegetational changes can be observed in the case of secondary succession, for instance after agricultural abandonment. Bare land is quickly occupied by early‐successional, fast‐growing ruderal plant species, which thrive under nutrient‐rich conditions. Over time and following the depletion of previously amended nutrients, these plants are gradually replaced by mid‐ or late‐successional plant species (Tilman, 1985; Walker *et al*., 2010).

These changes are driven by differences in plant traits that are plant species‐specific. For example, traits of early‐successional plants reflect their fast growth, enabling them to quickly exploit nutrients at a cost of a reduced defence against natural enemies, whereas mid‐ or late‐successional plants grow more slowly but are better defended against natural enemies (Grime, 1977; Bazzaz, 1979; Coley *et al*., 1985; Huston & Smith, 1987; van der Putten, 2003; Hakes & Cronin, 2012). Such contrasts among plants lead to differences in their adaptive strategies to allocate to growth or to defence in a given environment (Lind *et al*., 2013; de Vries *et al*., 2017; Züst & Agrawal, 2017). While studies have consistently shown that aboveground plant enemies (e.g. invertebrate herbivores) influence plant allocation of available resources to growth or defence (Stowe *et al*., 2000; Kessler & Baldwin, 2001; van der Putten, 2003), we still know little about how belowground communities and their interactions affect such strategies in plants. The lack of this knowledge is surprising given that soil microorganisms are known to drive plant performance and plant community dynamics (Berendsen *et al*., 2012; Philippot *et al*., 2013; Heinen *et al*., 2018).

These soil microorganisms profoundly change throughout succession such as plant communities. For example, bacteria‐dominated soils from agricultural and early‐successional plant communities become more fungi‐dominated as microbial succession progresses (Wardle *et al*., 2004; Maharning *et al*., 2009). Microbial community changes translate to altered microbial functioning, such as a decrease of pathogens with succession (Hannula *et al*., 2017). This functional change within soil microbes can directly feedback to plant performance. For example, vegetation dynamics can be facilitated by pathogen‐induced suppression of early‐successional plants, while better defended mid‐ and late‐successional plants are less affected by pathogens but promoted by mutualists (Hannula *et al*., 2017; Morriën *et al*., 2017; de Araujo *et al*., 2018). However, how complex microbial communities are linked to plant growth, plant defence and vegetation dynamics remains entirely unknown.

The tight link between plants and microbes at the level of growth and survival is omnipresent. Individual plant species, as well as plant communities, are influenced by interactions with soil bacteria (Lugtenberg & Kamilova, 2009; Berendsen *et al*., 2012), fungi (Rodriguez *et al*., 2009), protists (Gao *et al*., 2019; Xiong *et al*., 2020) and other soil organisms, such as soil invertebrates. Moreover, there are numerous complex interactions among all members of the microbiome, such as competition between bacteria and fungi (Bahram *et al*., 2018) and predator–prey interactions between bacteria and protists (Geisen *et al*., 2018) that affect microbial composition and functioning (Thakur & Geisen, 2019). These microbial interactions can affect plant performance in terms of their growth and also in terms of their defence (Pivato *et al*., 2009; van der Heijden & Hartmann, 2016; Thakur *et al*., 2019; Shen *et al*., 2020). How the individual roles of bacteria, fungi and protists, and interactions between and among these microbial groups in the soil food web vary among plant communities from different successional stages, and more importantly how they affect plant performance and their growth and defence strategies is not known.

Here we aimed to determine the roles of three major groups of soil microorganisms (bacteria, fungi and protists) and their interactions in contributing to the performance of early‐ and mid‐successional plant communities by measuring their growth, defence and the growth–defence relationship. We used grasses and forbs to obtain a broader understanding of the importance of microbial interactions on plant performance as these plant functional groups show fundamentally different growth and defence characteristics (Heinen *et al*., 2020a; Defossez *et al*., 2021) and also were shown to influence microbial communities in a functional group‐specific manner (Heinen *et al*., 2020b). In a glasshouse experiment, we inoculated plant communities with diverse communities of bacteria, fungi and protists in a full‐factorial way and determined effects on plant biomass, specific leaf area (SLA) and phenolics. We tested the hypothesis that distinct microbial groups differentially affect plant growth, defence compound production and growth–defence relationships (Hypothesis 1). Furthermore, we expected that competitive and trophic interactions between microbial groups alter plant responses (Hypothesis 2), and that these plant responses may further vary between early‐ and mid‐successional plant communities that differ between grasses and forbs (Hypothesis 3).

## Materials and Methods

### Soil sampling and preparations

In October 2017, we collected two independent soil samples at each of three early‐ and mid‐successional fields (Supporting Information Figs S1, S2). Early‐successional fields (all 3 yr after agricultural abandonment at time of soil collection) were Wageningse Eng Veld 3 (WA; 51°58.358′N, 5°41.295′E), Wegberm Paardenwei Telefoonweg 2 (WE; 52°00′9″N, 5°45′8″E) and Renkumse Heide (RE; 51°59′13.398″N, 5°44′43.73″E). Mid‐successional fields were Akker Reijerscamp (AK; 52°01.029′N, 5°47.394′E; 13 yr after abandonment at time of soil collection), Oud Reemst (OUD; 52°02.484′N, 5°48.527′E; 12 yr after abandonment at time of soil collection) and Reijerscamp (REY; 52°00.629′N, 5°46.971′E; 13 yr after abandonment at time of soil collection). We took two soil samples in a distance of 1 m for each of the two locations per field with a spoon from 5 cm below the surface. We then pooled the duplicate soil samples from each location (> 50 g) followed by sieving with a mesh size of 2 mm to remove stones and larger root pieces before isolation of the microorganisms.

### Isolation of microorganisms

#### Bacteria

We created a diverse bacterial community without the presence of other microorganisms as described in Rosenberg *et al*. (2009). In short, 20 g of sieved soil of each site was mixed with 20 ml Neff’s Modified Amoebae Saline (NMAS) buffer (Page, 1976) and filtered through coffee filters, followed by filtering through paper filters with decreasing mesh sizes with a final filtration step through 5.0 and 1.2 µm Isopore filters (Millipore). The resulting filtrate was mixed in 10% Nutrient Broth (NB)‐NMAS (Page, 1976). Cultivation flasks (Sarstedt, Nümbrecht, Germany) were stored at 20°C in the dark and investigated for bacterial growth as well as potentially contaminated cultures with protists or fungi under an inverted microscope (Leica Microsystems, Wetzlar, Germany) at ×200 and ×400 magnification. Contaminated flasks were refiltered with a 1.2 µm Isopore filter.

All 12 clean bacterial cultures (one from each of two independently taken samples at each of the six fields) were enriched by preparing overnight cultures in Tryptic Soy Broth (TSB) medium (CM0129; Oxoid, Basingstoke, UK) under constant shaking (90 rpm) at 35°C. Excess nutrients were then washed away by using duplicated centrifugation steps at 1690 ***g*** for 5 min in 50 ml NMAS and resuspending the dense bacterial communities in 15 ml NMAS. Optical densities of all cultures were adjusted to 0.1 before use in the glasshouse experiment.

#### Fungi

We obtained fungal cultures by diluting 20 g of each independent soil sample with sterile distilled water (1 : 10 ratio) in a 500 ml Erlenmeyer flask, shaking for 20 min at 100 rpm, again diluting with sterile water (1 : 10) and transferring 100 µl on a penicillin‐ and ampicillin‐containing water agar (1.0% agar, pH 6.7, ampicillin and penicillin: 250 mg l^−1^) in 9 cm Petri dishes. After 2 wk, 15 fungal colonies per Petri dish were transferred by cutting out single plugs and five were placed in the centre of three new universal fungal media plates (UF) containing 2% agar and 1.5% malt extract. Outer fungal pieces were transferred to new UF plates with a needle once the five fungal colonies expanded until the edge of the Petri dish was reached, a step that was repeated to ensure that only one fungal species was present per culture. A selection of fungi was made, based on morphologically and molecularly differentiated fungi (morphological grouping followed by sequencing the ITS region using primers ITS1–ITS4 as detailed in Methods S1) that were cultivated from both early‐ and mid‐successional soils. These were *Trichoderma hamatum* (three strains from early‐successional and three strains from mid‐successional soils (3/3))*, Mucor moelleri* (2/2)*, Mucor hiemalis* (1/1)*, Fusarium culmorum* (1/1)*, F. oxysporum* (1/1)*, Clonostachys rosea* (1/1)*, Trichoderma* spp. (1/1)*, Penicillium* sp. (1/1) and *Mortierella* spp. (1/1). All fungi were transferred to two new UF plates and stored at 20°C in the dark.

In preparation for the glasshouse experiment, six distinct fungal mixtures per successional stage were established, all of which containing the same nine species but with variable mixes of strains for both early‐ and mid‐successional treatments (see Methods S1, Extended Table 1 for details). For that we pipetted 5 ml sterilized water to fungal plates to collect spore and hyphal parts with a cell scraper. For each fungal culture, we pooled all fungal suspensions obtained from the replicated Petri dishes used to increase fungal biomass for inoculation. We adjusted the fungal biomass added by standardizing the optical density of each culture to OD_450_ of 0.1. We tested successful establishment of all fungi by adding these to agar plates. Last, we mixed 5 ml of each fungal culture with the other eight cultures per successional stage in a 50 ml tube.

#### Protists

A modified liquid aliquot method was used to isolate protists from the soils of each field site (Geisen *et al*., 2014). In short, 1 g of sieved soil was homogenized in 200 ml NMAS by initial vigorous manual shaking and subsequent shaking at 100 rpm for 10 min. The suspension was inverted and shaken vigorously for 10 s and left to settle for 5 min. From the centre of the suspension, 5 µl were transferred to individual wells of a 96‐well plate (Greiner Bio‐One GmbH, Frickenhausen, Germany) prefilled with 10% NB‐NMAS to allow slow bacterial growth. Plates were sealed with Parafilm and stored in the dark at 20°C. After 2 wk, each well was microscopically examined for microbial growth and those wells where only protists were growing were selected for subsequent cultivation by transferring them into 6 cm Petri dishes also prefilled with 10% NB‐NMAS. All individual protist cultures were kept in order to cultivate as many protists with distinct morphology (indicating that these represent different taxa) as possible. Of those protists that were chosen for the experimental use, 1 ml of each culture was transferred to three new 6 cm Petri dishes filled with 10% NB‐NMAS and grown for 3 d at 20°C. Then, the protist solution from all three replicates were pooled and washed twice with 50 ml NMAS by centrifugation at 152 ***g*** for 10 min and resuspending the pallet in 15 ml NMAS. Six protist mixes were created for inoculation into the experiment. Each mix contained individual species of amoebo‐flagellates from the Glissomonadida, ciliates of the genus *Colpoda*, amoeba of the genus *Acanthamoeba* and of the class Heterolobosea (Methods S1, Extended Table 2). Three distinct mixes containing a random mix of four protist taxa were established for each of the two successional stage (see Methods S1 for details). Numbers of protists were estimated under a microscope and adjusted to 1000 ml^−1^ before inoculation.

### Glasshouse experiment

We chose three grass and two forb species of both early‐ and mid‐successional stages based on previous work in the successional chronosequence from which soils were collected and more recent own observations (Table 1; van de Voorde *et al*., 2011). As grasses all belong to the same family (Poaceae), we balanced the design phylogenetically, by also choosing forb species from a single family (Asteraceae). Based on own observations, Asteraceae are among the most represented families of forbs in the chronosequence, in terms of both species number and abundances.

**Table 1:** Detail of plant species used in the glasshouse experiment.

Species	Abbreviations	Successional stage	Functional group	Family
*Jacobaea vulgaris* L.	JV	Early	Forb	Asteraceae
*Hypochaeris radicata* L.	HR	Early	Forb	Asteraceae
*Leucanthemum vulgare* L.	LV	Mid	Forb	Asteraceae
*Taraxacum officinale* L.	TO	Mid	Forb	Asteraceae
*Alopecurus pratensis* L.	AP	Early	Grass	Poaceae
*Poa trivialis* L.	PT	Early	Grass	Poaceae
*Holcus lanatus* L.	HL	Early	Grass	Poaceae
*Festuca rubra* L.	FR	Mid	Grass	Poaceae
*Poa pratensis* L.	PP	Mid	Grass	Poaceae
*Dactylis glomerata* L.	DG	Mid	Grass	Poaceae

Seeds (all species were bought at Cruydt‐Hoeck, Nijeberkoop, the Netherlands, except *Jacobaea vulgaris*, for which seeds were collected locally in 2014) were sterilized in 0.4% sodium hypochlorite solution for 3 min, then rinsed with sterile distilled water (H_2_O_dest_), sterilized in 96% ethanol for 5 min and finally rinsed in H_2_O_dest_. Seeds were germinated in sterile soil or glass beads in a climate chamber (24°C, 16 h : 8 h, day : night, RV 70%). Upon germination, the early‐germinating species were stored in a cold room (4°C, 16 h : 8 h, day : night, RV 80%) until the later‐germinating species had also germinated, to ensure seedlings were planted at the same growth stage (i.e. similar height).

In total 192 (10 × 10 × 11 cm, 1 l) pots were filled with 1050 g sterile soil (sterilized with 8 kGy gamma radiation; Isotron, Ede, the Netherlands). Soil physicochemical properties (‘De Mossel’, Ede, the Netherlands) are described in Jeffery *et al*. (2017). In short, it is a holtpodzol, sandy loam (94% sand, 4% silt, 2% clay, *c.* 5% organic matter, 5.2 pH, 2.5 mg kg^−1^ N, 4.0 mg kg^−1^ P, 16.5 mg kg^−1^ K). The selected species differ strongly in their aboveground biomass cover, which differed most strongly between the two functional groups, with forbs generally having a higher aboveground density than grasses. To account for these differences, plant communities were planted with (visually) similar aboveground cover densities. To achieve this, species were planted in two densities: forb communities consisted of two individuals of two species, whereas grass communities consisted of four individuals of three species (see Fig. S1). For the respective microbial treatments, the same concentration of cells was added in 1 ml fungal, 1.5 ml bacterial and 2 ml protist mix. The same amount of the respective microbial medium was sterilized by autoclaving (20 min, 121°C, 1500 kPa) and added to plant communities not receiving the respective life‐inoculum to balance out potential effects by adding nutrients or changes in soil moisture. Mixes of living or sterilized protists were added 4 d after initiating the experiment to ensure that sufficient microbial prey had formed for them to feed upon. We note that the microbial communities are used as models (although collected from field soils) that do not simulate realistic microbial communities in the field, as those are variable over time and also orders of magnitude more complex. Nevertheless, microorganisms used in our study represent some of the most abundant soil microbial groups, and our experiment aims to provide a causal link between microbial group interactions and plant performance.

**Fig. 1:**
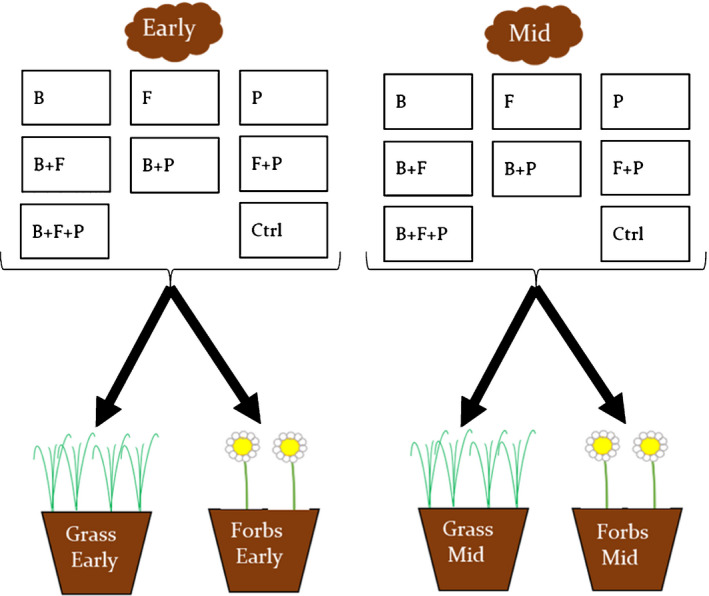
Conceptual design scheme illustrating the full‐factorial microbial treatments consisting of bacteria, fungi, protists and their interactions. Microbes were isolated from early‐ or mid‐successional soils and inoculated to either four different grasses or two forb species commonly growing in the respective successional stage. B, bacteria only; F, fungi only; P, protist only; B + F, bacteria and fungi together; B + P, bacteria and protist together; F + P, fungi and protist together; B + F + P, bacteria; fungi and protist together; C, sterilized soils.

Together, the experimental setup was a full factorial design to include the respective microbial groups (bacteria, fungi and protists) for the two plant successional stages separately for grasses and forbs. We used early‐successional grasses, early‐successional forbs, mid‐successional grasses, and mid‐successional forbs, and each of them received eight microbial treatments (Figs 1, S2) replicated six times, yielding 192 pots (4 × 8 × 6). These were randomly placed in the glasshouse for 8 wk (16 h : 8 h, 21°C :  16°C, day : night, 60% relative humidity; high‐pressure sodium Son‐T, 600 W Philips GP lamps (Amsterdam, the Netherlands) and sterile water supply twice per week by weighing to the initial 1050 g of soil). Plants were randomized weekly, placed on individual watering dishes and watered with small glass beakers to avoid cross‐contamination through water splash.

After 8 wk, we harvested shoot material per species from each of the experimental pots. A fully expanded healthy leaf was sampled from a randomly selected individual per species within each community to determine SLA. The leaves were scanned (Epson 4990; Seiko Epson Corp., Suwa Nagano, Japan) and the surface area measured using WinFolia 2016 (Regent Instruments, Quebec, QC, Canada; https://regentinstruments.com), oven‐dried at 60°C and then weighed. Shoot samples were combined per species per experimental pot and shock‐frozen in liquid nitrogen followed by freeze‐drying for 3 d before weighing shoot dry biomass. Roots were thoroughly washed over a fine sieve to remove soil particles and dried at 80°C to determine root dry biomass.

### Phenolic acid analysis

We extracted phenols from plant leaves as a measurement of constitutive plant defence given that no herbivores were used in our study (Daayf *et al*., 2012). The reason for using shoots, but not roots, was that root networks could not be disentangled and traced to plant species as they were heavily intertwined. Also, recent work suggested that belowground microbial communities can affect aboveground plant defences, often even outweighing induced defence responses to, for instance, chewing herbivores (Zhu *et al*., 2018; Ristok *et al*., 2019; Huberty *et al*., 2020). Also soil microbial‐mediated aboveground plant responses affect aboveground herbivory (Heinen *et al*., 2020b; Howard *et al*., 2020), and hence are a robust way to test plant growth–defence relationships. Phenolic compounds were extracted using a methanol (MeOH 70%) extraction procedure. For this, dry leaf material was ground using Tissue Lyzer II (Qiagen) at maximum speed for 2 min. Two extractions from 20 mg ground material were performed for each sample. For the first extraction, 1 ml 70% MeOH was added to each sample, vortexed for 30 s, ultrasonicated at 20°C for 30 min and centrifuged (Sartorius, Gottingen, Germany) at 9168 ***g*** for 10 min. The supernatant was transferred to a clean centrifuge tube. The extraction was repeated and supernatants combined to increase the extraction efficiency. The extract was then filtered with a 13 mm syringe filter (VWR International, Darmstadt, Germany) and a 0.2 µm polytetrafluoroethylene syringe (Henske Sass Wolf GmbH, Tuttlingen, Germany). Extracts were stored at −20°C.

Phenolic acids were separated by injecting 5 µl sample into a Thermo Hypersil gold column 250 × 4.6 mm (ThermoFisher Scientific, Waltham, MA, USA) at 22°C in a HPLC:Ultimate 3000 (ThermoScientific, MA, USA) equipped with a UV diode array detection. The mobile phase consisted of 95% of 0.5% phosphoric acid (5 ml l^−1^ MilliQ H_2_O) and 5% acetonitrile, and the flow rate was 1.0 ml min^−1^. Simultaneous monitoring of peaks was performed at detection wavelengths of 350, 278, 300 and 370 nm.

High‐performance liquid chromatography raw data were analysed with Chromeleon™ chromatography data system (CDS) software from ThermoFisher Scientific. We first identified known compounds based on 20 known reference compounds, of which six (chlorogenic acid, epicatechin, rutin, p‐coumaric acid, caffeic acid and myricetin) were present in our samples, all being potential plant defence compounds against pathogens (e.g. Matern & Kneusel, 1988; Leiss *et al*., 2009; Li *et al*., 2009; Yamaji & Ichihara, 2012; Yang *et al*., 2016). In addition, we found six unknown compounds, which were analysed based on their peak area.

### Statistical analysis

The variation in growth and defence variables measured at the plant species level (shoot biomass, SLA, total phenolics) were analysed using two‐way ANOVA mixed‐effects models with microbial treatments and plant succession type as two fixed effects and plant species as the random intercept. For the pot‐level data (i.e. at the plant community level; shoot biomass, root biomass, shoot : root ratio), we used two‐way ANOVA with microbial treatments and plant succession type as fixed effects, and with no random effects. We used Tukey *post hoc* tests to examine the group‐specific differences when either the fixed effects or their interactions were statistically significant (*P* < 0.05). Model assumptions (e.g. the homogeneity of variance and normality of residuals) were tested visually for each model. Some response variables were log‐transformed to meet the model assumptions and these occurrences are indicated in Table 2 and figure legends. All our statistical models were run separately for communities of grasses and forbs.

**Table 2:** Responses of grass and forb growth and defence variables explained by microbial treatments and plant succession type.

	Fixed effects						Random effect
	Microbial treatments (M)		Plant succession type (P)		M × P		
	*F*‐value_df_	*P*‐value	*F*‐value_df_	*P*‐value	*F*‐value_df_	*P*‐value	Plant species variance (SD)
Grass							
Shoot biomass	0.52_7,266_	0.81	0.01_1,4_	0.92	0.78_7,266_	0.59	0.57 (0.75)
Root biomass (log‐transformed)	1.06_7,80_	0.39	1.20_1,80_	0.27	1.90_7,80_	0.08	
Shoot : root biomass (log‐transformed)	1.31_7,80_	0.25	2.55_1,80_	0.11	**2.19_7,80_**	**0.04**	
Specific leaf area (log‐transformed)	1.61_7,265_	0.13	2.08_1,4_	0.22	**3.95_7,265_**	**< 0.001**	0.02 (0.15)
Total phenolics (log‐transformed)	0.86_7,249.07_	0.53	2.44_1,4_	0.19	0.86_7,249.07_	0.53	0.32 (0.56)
Forbs							
Shoot biomass	0.09_7,174_	0.99	0.22_1,2_	0.67	0.56_7,174_	0.56	0.11 (0.33)
Root biomass	1.20_7,80_	0.31	**29.67_1,80_**	**< 0.001**	0.34_7,80_	0.93	
Shoot : root biomass (log‐transformed)	2.06_7,80_	0.05	**77.19_1,80_**	**< 0.001**	0.93_7,80_	0.48	
Specific leaf area (log‐transformed)	1.35_7,174_	0.22	< 0.01_1,2_	0.99	0.95_7,174_	0.46	0.07 (0.28)
Total phenolics (log‐transformed)	**3.31_7,157_**	**< 0.01**	0.02_1,2_	0.89	0.98_7,157_	0.44	0.38 (0.62)

Bold *F*‐ and *P*‐values are statistically significant (*P* < 0.05). df, degrees of freedom, SD, standard deviation.

The variation in composition of 12 different phenolic compounds (six known and six unknown) was further analysed using principal component analysis (PCA), from which we obtained PCA axis scores. Given that we detected ‘horseshoe or arch’ effect in our raw phenolic data as a result of the absence and low levels of detection of many phenolic compounds, we used Hellinger transformation as a potential remedy to this (Legendre & Gallagher, 2001; Zuur *et al*., 2007). We ran PERMANOVA tests to examine whether variation in phenolic composition was explained by plant succession type and soil microbial treatments as well as the interaction between the two. Finally, we used the scores of the first and second PCA axes (as they two explained most of the variation in phenolic composition) as a variable to represent overall defence variation in plant communities. We then associated the first and second PCA axis scores with the shoot biomass using simple linear regression to examine how growth–defence relationships changed across soil microbial treatments and plant succession type.

We ran all statistical tests in R v.3.5.2 (R Core Team, 2019). All mixed‐effects models were run with the lme4 package (Bates *et al*., 2015). The *F*‐values from the mixed‐effects models and the estimation of their degrees of freedom (based on Kenward–Roger approximation) were obtained using the lmertest package (Kuznetsova *et al*., 2017). The post‐hoc Tukey tests were performed using the multcomp package (Hothorn *et al*., 2008). PCA and PERMANOVA test were performed using the vegan package (with the ‘adonis’ function; Oksanen *et al*., 2017). The *R* ^2^ values of linear models were adjusted *R* ^2^ and their statistical significance were obtained from the broom package (Robinson & Hayes, 2018). Model diagnostics were carried out using the performance package (Lüdecke *et al*., 2019).

## Results

**Fig. 2:**
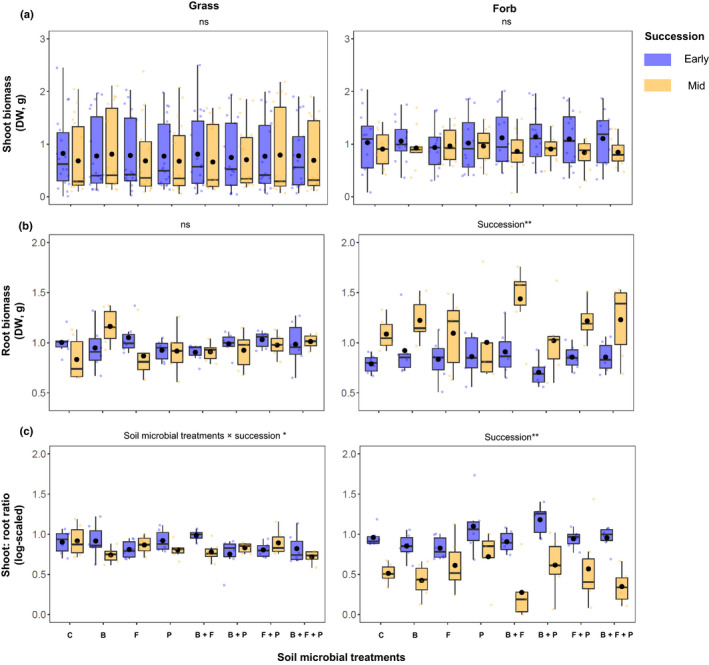
Shoot (a) and root (b) biomasses, and shoot : root ratio (c) of early‐ (blue boxplots) and mid‐successional (yellow boxplots) grasses (left panels) and forbs (right panels). C, control; B, bacteria; F, fungi; P, protists. Statistically significant differences are highlighted at the top in each panel (asterisks represent level of significance: *, *P* < 0.05; **, *P* < 0.01; ns, nonsignificant differences). Circles inside boxplots represent the mean, horizontal lines median values. The biomass share of individual plant species is provided in Supporting Information Fig. S3. Number of observations for grass communities per successional type per microbial treatment = 18; number of observations for forb communities per successional type per microbial treatment = 12.

The community shoot biomass of the grass and the forb communities was unaffected by microbial treatment or successional type, or their interaction (*P* > 0.05; Table 2; Fig. 2a). The community root biomass was significantly higher in mid‐successional forbs than in early‐successional ones irrespective of microbial treatments (*F* = 29.67, *P* < 0.001, Table 2; Fig. 2b). We did not observe changes in root biomass in grasses, nor did microbial treatments influence the community root biomass in grasses (*P* > 0.05, Table 2). The shoot : root ratio of forb communities was significantly lower in mid‐successional than in early‐successional communities, irrespective of microbial treatments (*F* = 77.19, *P* < 0.001; Table 2; Fig. 2c), probably driven by differences in the root biomass. Microbial treatments and successional type interacted in affecting the shoot : root ratio of grass communities (*F* = 2.19, *P* = 0.04; Table 2; Fig. 2c), potentially driven by an increased shoot : root ratio in early successional communities in bacterial‐only and bacterial–fungal mixed treatments. However, *post hoc* tests did not reveal any group‐specific differences.

**Fig. 3:**
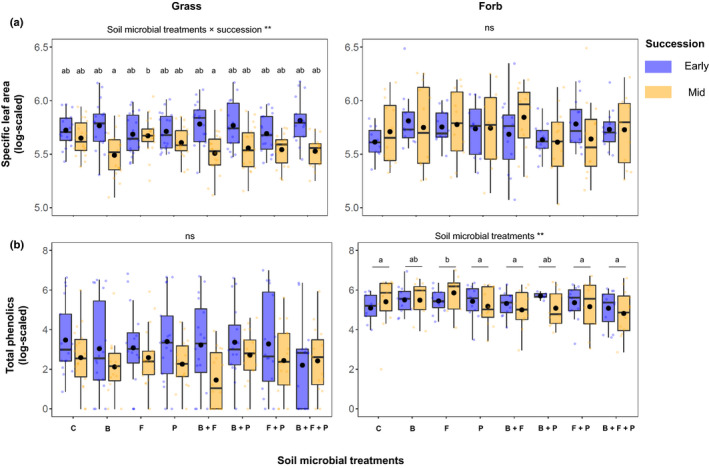
Specific leaf area (a) and total concentration of phenolics (b) in early‐ (blue boxplots) and mid‐successional (yellow boxplots) grasses (left) and forbs (right). C, control; B, bacteria; F, fungi; P, protists. Statistically significant differences are highlighted at the top in each panel (asterisks represent level of significance: *, *P* < 0.05; **, *P* < 0.01; ns, nonsignificant differences). *Post hoc* results based on *P* < 0.05 are shown as letters above the boxplots. Circles inside boxplots represent the mean, and horizontal lines represent median values. Number of observations for grass communities per successional type per microbial treatment = 18; number of observations for forb communities per successional type per microbial treatment = 12.

Microbial treatments and plant succession type interactively affected SLA in grass communities (*F* = 3.95, *P* < 0.001; Table 2; Fig. 3a). This interaction effect was mainly driven by the greater SLA in fungal‐only treatments than bacteria‐only and the bacterial–fungal mixed treatments of mid‐successional grass communities (Fig. 3a). Microbial groups did not affect SLA of forb communities (*P* > 0.05; Fig. 3a).

**Fig. 4:**
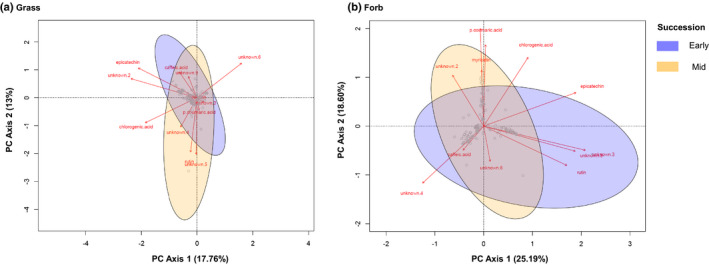
Biplots based on principal component analysis (PCA) of 12 phenolic compounds detected in grass (a) and forb (b) communities. Based on PERMANOVA results, we show the grouping between early‐ and mid‐succesional plant communities for both grasses and forbs.

Total phenolics did not vary in grass communities (*P* > 0.05; Table 2; Fig. 3b). By contrast, microbial treatments significantly explained the variation in total phenolics in forb communities independent of plant succession type (*F* = 3.31, *P* < 0.01; Table 2; Fig. 3b), mainly owing to higher total phenolics in fungi‐only treatments (Fig. 3b). The first PCA axis based on 12 different phenolics in grass communities explained 17.76% of the total variation, whereas the second axis explained 13% of the total variation (Fig. 4a). In forb communities, the first axis explained 25.19%, and the second axis 18.60% of the total variation in phenolics (Fig. 4b). Moreover, we found that plant succession type significantly explained the variation in phenolic composition for both grasses (*F* = 22.04, *P* = 0.001) and forbs (*F* = 5.41, *P*‐0.006). We found no effects of microbial treatments (grasses, *F* = 1.41, *P* = 0.09; forbs, *F* = 0.41, *P* = 0.99) or interaction with plant succession (grasses, *F* = 0.73, *P* = 0.82; Forbs, *F* = 1.15, *P* = 0.30).

Different plant functional groups shifted their phenolic compound composition in distinct directions, and this variation was associated with notable shifts in particular compounds. In grasses, PC1 scores were negatively correlated with epicatechin, unknown compound 2, and chlorogenic acid, whereas PC2 was negatively correlated with rutin and unknown compounds 4 and 5 (Fig. 4a). In forbs, PC1 scores were positively correlated with epicatechin, rutin and unknown phenolic compounds 3 and 5, whereas PC2 scores were positively correlated with p‐coumaric acid and myricetin (Fig. 4b).

**Fig. 5:**
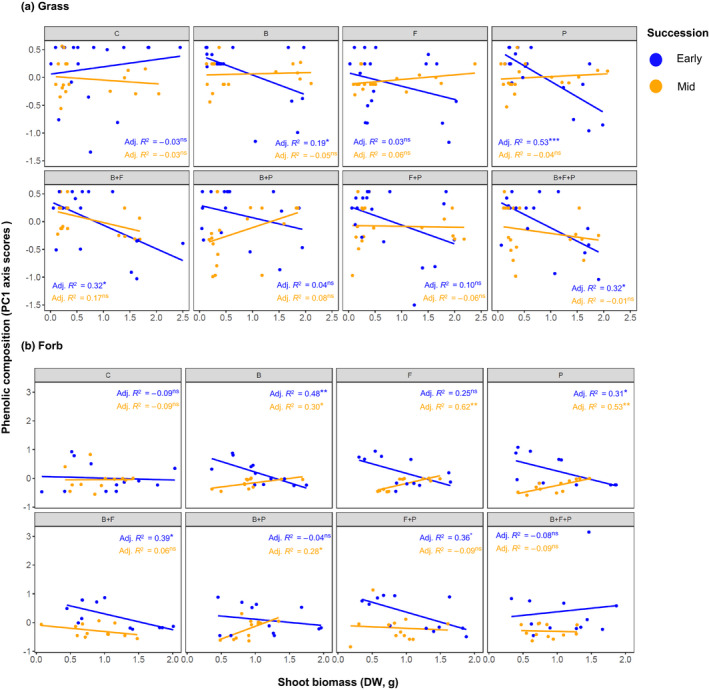
Composition of phenolics as expressed by PC1 scores against shoot biomasses (in g) in grasses (a) and forbs (b). C, control; B, bacteria; F, fungi; P, protists. Statistically significant correlations are indicated with asterisks (level of significance: *, *P* < 0.05; **, *P* < 0.01; ns, nonsignificant differences). Number of observations for grass communities per successional type per microbial treatment = 18; number of observations for forb communities per successional type per microbial treatment = 12.

The relationships between PCA axes scores (based on the variation in 12 different phenolic compounds) and plant shoot biomass varied across microbial treatments and plant succession types in both grass and forb communities (Figs 5a,b, S4). A consistent pattern in these correlations was that there were several instances of opposite relationships between PC axis 1 scores (as a proxy of phenolic composition) and shoot biomass in early‐ and mid‐successional plants that were most pronounced in forbs (Fig. 5b). For instance, shoot biomass was negatively correlated with variation in phenolic composition in early‐successional forb communities, but this relationship was the opposite in mid‐successional forb communities when bacteria or protists were present (Fig. 5b). Interestingly, while we found some instances of the same negative relationship in early‐successional grass communities (e.g. also in bacteria‐ or protist‐only treatments), there was not a single significant correlation between variation in phenolic composition (based on both PCA axes) and shoot biomass of mid‐successional grasses with any microbial treatment (Figs 5a, S4A). The presence of complex interactions, such as when all three microbial groups were present in the soil, removed shoot biomass–phenolic compositional variation relationships in both types of forbs (Fig. 5b).

## Discussion

We show that individual microbial groups and their complex interactions within the rhizosphere microbiome can affect the relationships between growth (measured as plant biomass) and defence (measured as phenolic compound composition) in plant communities, suggesting microbial‐mediated shifts in growth–defence relationships. While these results partly confirm Hypothesis 1, microbial treatments did not strongly influence plant growth and defence when analysed separately. The effects of competitive and trophic microbial interactions between soil microbes were also pronounced in the growth–defence relationship, but hardly so for growth and defence alone – thus in partial agreement with Hypothesis 2. Most of these effects differed between plant functional group and between plant successional stage, which is in agreement with Hypothesis 3.

Tight links between free‐living soil microorganisms and plants are well known. For example, plants shape their microbiome through root exudation (Bulgarelli *et al*., 2012; Lundberg *et al*., 2012) partly to defend against antagonists (Agrawal & Karban, 1999). Thereby, root exudates directly influence plant performance (Lundberg *et al*., 2012; van der Putten *et al*., 2013; Heinze *et al*., 2020). In turn, microorganisms change plant performance, including plant defence compounds, by altering nutrient availability in soils or by colonizing roots (Wurst *et al*., 2010; Tyc *et al*., 2017). We report here unique shifts of secondary metabolite production in a microbial group‐specific and even a microbial interaction‐specific manner that were especially pronounced in forb communities. Notably, mainly the presence of fungi removed the positive growth–defence correlation in mid‐successional forbs found with bacteria, fungi and protists alone, but only so in presence of other microorganisms. This suggests that fungal interactions with other microorganisms could diversify phenolic compounds in plants (Schmidt *et al*., 2015). Such a diversification might help the plant to fight off the many facultatively antagonistic fungi that can directly penetrate plant roots (Rodriguez *et al*., 2009) such as *Fusarium* spp. included in our study. Fungal‐induced defence shifts in interaction with other microbes might not even negatively affect plant growth, as recently suggested (Bastías *et al*., 2021). Indeed, many of the fungi we included are potential root endophytes (Geisen *et al*., 2017a), underlining the likely importance of not only fungal pathogens but endophytes in affecting plant performance such as shifting growth–defence tradeoffs (Bastías *et al*., 2021). We show here that these effects are plant functional group and successional stage‐dependent. Even more strikingly, fungal effects are determined by the presence of bacteria and protists, probably through competitive (Schmidt *et al*., 2015; Bahram *et al*., 2018) and trophic (Geisen *et al*., 2016) interactions.

Early successional forbs showed a contrasting pattern to mid‐successional forbs in terms of how soil microbial interactions affected the relationship between shoot biomass and variation in phenolics composition. For example, we often found a negative relationship between shoot biomass and variation in phenolics composition in early‐successional forbs, and the same was true for early‐successional grasses. Such a negative relationship can be expected for fast‐growing species (that many early‐successional plants are relative to mid‐successional plants) as they usually invest more in growth and less in defence. Here, the presence of microbial predator–prey interactions (protist–bacteria interaction, and also protist–fungi interactions) shifted the negative correlation between shoot biomass and phenolic composition that was present in other microbial treatments. Soil microbial predators can catalyse microbiome shifts that are likely to affect plant resource allocation (Thakur & Geisen, 2019). Protist predators are known to change soil bacteria in many ways; protists change bacterial community composition, activity and induce changes in microbial‐produced secondary metabolites (Alphei *et al*., 1996; Rosenberg *et al*., 2009; Henkes *et al*., 2018), patterns that might be of similar importance in protist–fungi interactions (Geisen *et al*., 2016). These predator–prey interactions can be mediated by diverse microbial secondary metabolites (Jousset, 2012; Schulz‐Bohm *et al*., 2017) which can alter secondary metabolite production, nutrient allocation and morphological changes in plants (Bonkowski & Brandt, 2002; Rosenberg *et al*., 2009; Koller *et al*., 2013; Scherlach *et al*., 2013; Tyc *et al*., 2017).

In addition to differences in successional stages, we found distinct responses in growth–defence relationships between plants from different functional groups that were most pronounced in forbs. Distinct patterns in the two functional groups can be explained by various factors, such as differences in (root) traits (Tjoelker *et al*., 2005) and root exudation (Herz *et al*., 2018; Dietz *et al*., 2020), which might shift microbial interactions in the rhizosphere (Kos *et al*., 2015; Heinen *et al*., 2018). Another explanation can be found in the different chemical defences used by grasses and forbs, with grasses having a less diverse bouquet of secondary metabolites than forbs (Tscharntke & Greiler, 1995; Defossez *et al*., 2021). Also, grasses, as well as their associated herbivores, are generally less responsive to changes in soil microbial communities than forbs and their associated herbivores (Heinen *et al*., 2020a, 2020ba,b), which also suggests that inducible defences may play a smaller role in grasses than in forbs. Instead, grasses may rely more on constitutive defences, including structural defences or silicates (McNaughton & Tarrants, 1983).

We note that the interpretation of the observed microbial‐driven shifts in correlation between plant shoot and phenolic composition (based on PC axis scores) is not straightforward. A simple interpretation of significant correlations between the two would be that shoot biomass and variation in phenolic composition depend on each other. As we were interested in understanding the role of soil microorganisms in driving plant growth–defence relationships, we present two plausible interpretations of shifts in these correlations: positive correlation would mean that plants are able to diversify their phenolic defence (in terms of higher variability in the production of 12 phenolic compounds) and possibly do so by producing greater biomass; and negative correlation implies cost on either growth or on the diversification of phenolics composition. We provide examples of these interpretations based on plant functional group and their successional stage in our study. Our results further imply that the variation in phenolic compounds in the PC axes can be explained by shifts in the abundances of specific phenolic compounds, notably epicatechin, chlorogenic acid, rutin and p‐coumaric acid, all of which have been shown to have defensive properties (Matern & Kneusel, 1988; Izaguirre *et al*., 2007; Leiss *et al*., 2009; Li *et al*., 2009; Yamaji & Ichihara, 2012; Yang *et al*., 2016; Kundu & Vadassery, 2019).

Our study was designed as a proof‐of‐concept pot experiment under controlled glasshouse conditions that comes with caveats. For instance, the observed shifts in the relationship between shoot biomass and phenolics composition do not imply any causative changes driven by any particular group of microorganisms, meaning that we cannot assume that a given shift leads to increased (or decreased) defence responses at the expense of plant growth. Such shifts in correlation, however, provide new insights into the potential role of certain groups of soil microorganisms and their interactions in shifting a plant’s growth and defence strategy. Indeed, the microbial interactions and how they affect vegetation may not be generalized to other microbes from the same group, or to more complex microbial communities present in natural soils. Potential invasions from diverse airborne microbes are to be expected in our pots (Geisen *et al*., 2017a; de Groot *et al*., 2021), which might reduce effect sizes such as removing microbial treatment effects on plant biomasses. As invasions are random and because microbial priority effects play a key role in determining plant performance (Ke & Wan, 2020; Xiong *et al*., 2020), we assume that all observed effects are a result of our initial microbial treatments. Another important notion is that the observed microbial effects on the two plant functional groups cannot be generalized for all plant species in these and other functional groups. Importantly, defence compound identity could show different responses depending on the group of compounds, and might vary between shoots and roots (Gargallo‐Garriga *et al*., 2014). We intended to study shoot metabolites to investigate nonlocal tissue‐specific responses, which have recently been shown to respond to soil microbial composition (Ristok *et al*., 2019; Huberty *et al*., 2020). Indeed, differences in the composition of root phenolics were often shown to have a profound importance for plant performance (Meier *et al*., 2008), and it is likely that the interacting soil microorganisms will induce even stronger metabolic changes in roots. Furthermore, we used communities of grasses and forbs that differed in species number and abundances to balance aboveground biomass, and although we chose a phylogenetically balanced design of one dominant plant family in each group, it could clearly be argued that Asteraceae do not represent the wide diversity of forbs. However, including a broader range of forbs compared with a single family of grasses probably increases the variation in observed forb defences and would potentially inflate differences between functional groups. We argue that including a wider range of plant species and functional groups is expected to increase the effect sizes and show even more clearly the importance of microbial interactions for plant performance.

In summary, our study provides a novel and unique approach to manipulate microbial groups in simple soil communities that controls for differences in species composition between successional stages by investigating species from similar taxa in both early‐ and mid‐successional treatments. Strikingly, these soil microbial communities exhibited different effects depending on plant communities, which shows that even closely related microbial species can functionally differ and interactions among microbial groups can potentially shift the relationship between plant biomass and phenolic compositional variations consistently in forbs and occasionally in grass communities. These microbially mediated plant growth–defence relationships might have far‐reaching consequences for plant fitness in natural settings. As such, our study profoundly expands previous studies on single microbe–plant species interactions (Geisen *et al*., 2017b) to show that model species approaches might provide oversimplified answers to questions related to microbe–microbe–plant interactions and that more complex studies are needed to study plant–microbe interactions.

## Author contributions

SG conceived the study with input from RH and MPT. SG, RH, TvL, FCtH and MPT set up and performed the experiment. SG, EA, RH and MPT gathered all data, which were analysed by MPT. SG, RH and MPT wrote the manuscript and all other authors helped in revising it.

## Supporting information


**Fig. S1** Design of plants in a pot.
**Fig. S2** Design of the experimental scheme focusing on microbial treatments.
**Fig. S3** Shoot biomasses of individual grass species and forb species.
**Fig. S4** Composition of phenolics as expressed by PC2.
**Methods S1** Detailed information on the establishment of fungal and protist cultures.
Please note: Wiley Blackwell are not responsible for the content or functionality of any Supporting Information supplied by the authors. Any queries (other than missing material) should be directed to the *New Phytologist* Central Office.

## Data Availability

All data are directly attached to this paper.
