# Supplementary material for: Contrasting effects of soil microbial interactions on growth–defence relationships between early‐ and mid‐successional plant communities
Source: New Phytol. 2021 Aug 19;233(3):1345–57. doi: 10.1111/nph.17609 (PMC9292498; doi:10.1111/nph.17609)
Supplement: Supplementary file 1 — Fig. S1 Design of plants in a pot. Fig. S2 Design of the experimental scheme focusing on microbial treatments. Fig. S3 Shoot biomasses of individual grass species and forb species. Fig. S4 Composition of phenolics as expressed by PC2. Methods S1 Detailed information on the establishment of fungal and protist cultures. Please note: Wiley Blackwell are not responsible for the content or functionality of any Supporting Information supplied by the authors. Any queries (other than missing material) should be directed to the New Phytologist Central Office. [file NPH-233-1345-s001.pdf]

New Phytologist

***New Phytologist* Supporting Information**

Article title: Contrasting effects of soil microbial interactions on growth-defence relationships between early- and mid-successional plant communities
Authors: Stefan Geisen, Robin Heinen, Elena Andreou, Teun van Lent, Freddy C. ten Hooven, Madhav P. Thakur
Article acceptance date: 01 July 2021

The following Supporting Information is available for this article:

Fig. S1 Design of plants in a pot. Above two are grasses, below are forbs. The left two pots are early-successional plants, on the right are mid-successional plants. Each replicate has the same design. Abbreviations as in Table 1

Fig. S2 Design of the experimental scheme focusing on microbial treatments.

Fig. S3 Shoot biomasses of individual grass species (A) and forb species (B).

Fig. S4 Composition of phenolics as expressed by PC2 Scores against shoot biomasses (in gram) in grasses (A) and forbs (B).

New Phytologist

Methods S1

Here, supplementary information is found to help to understand the experimental setup (Figs. S1 and S2), additional results (Figs. S3 and S4) and additional methods (

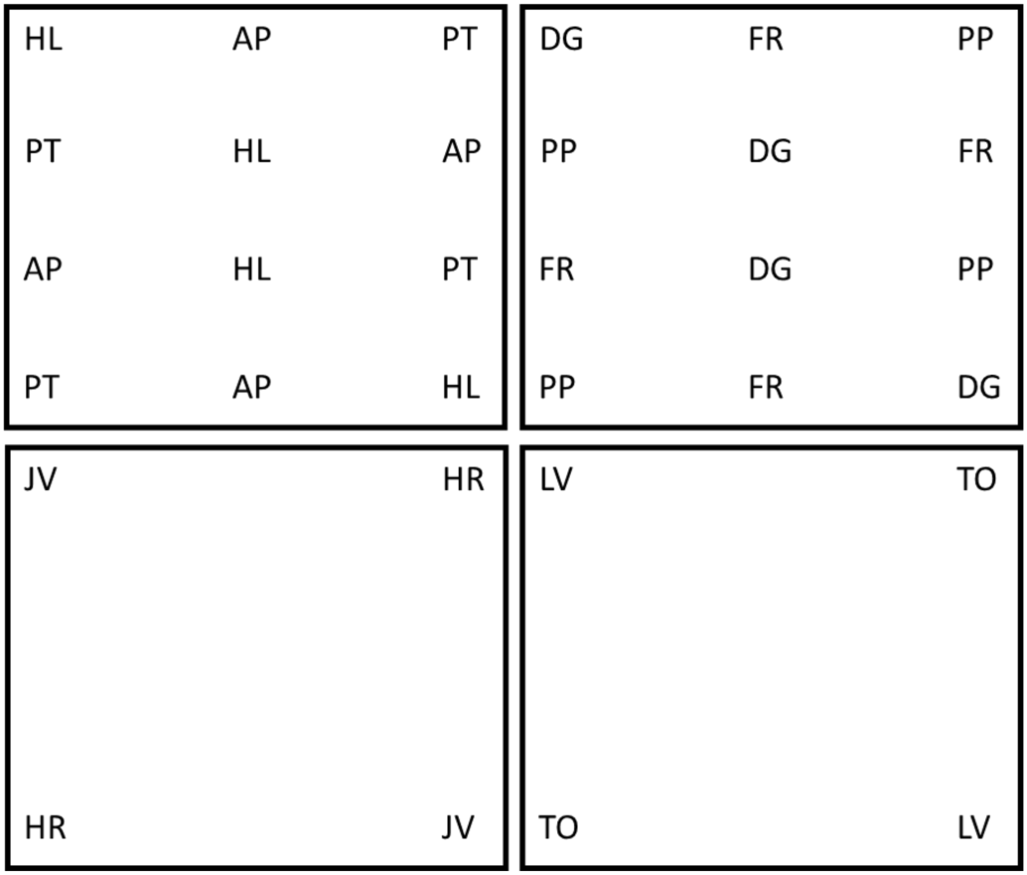

Fig. S1 Design of plants in a pot. Above two are grasses, below are forbs. The left two pots are early-successional plants, on the right are mid-successional plants. Each replicate has the same design. Abbreviations as in Table 1

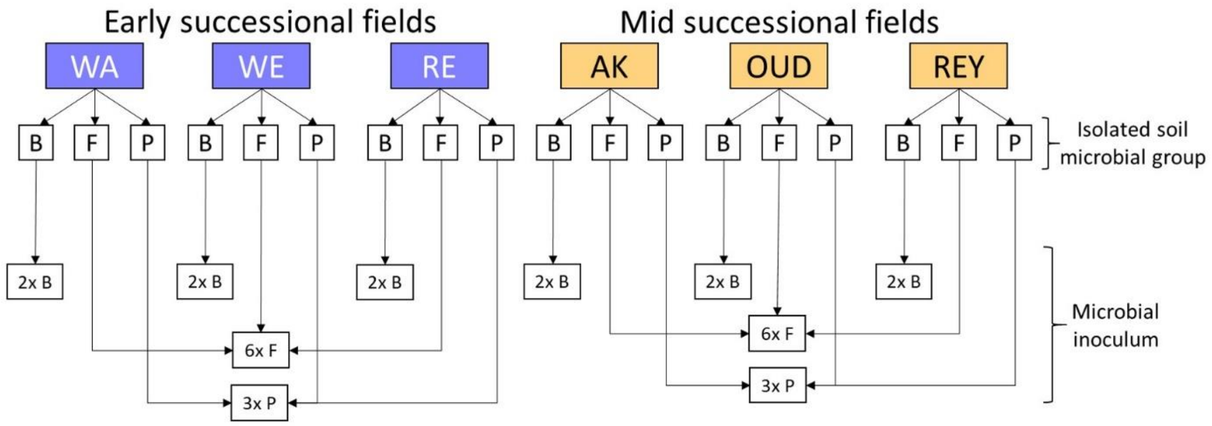

Fig. S2 Design of the experimental scheme focusing on microbial treatments. C: Control; B: Bacteria; F: Fungi; P: Protists.

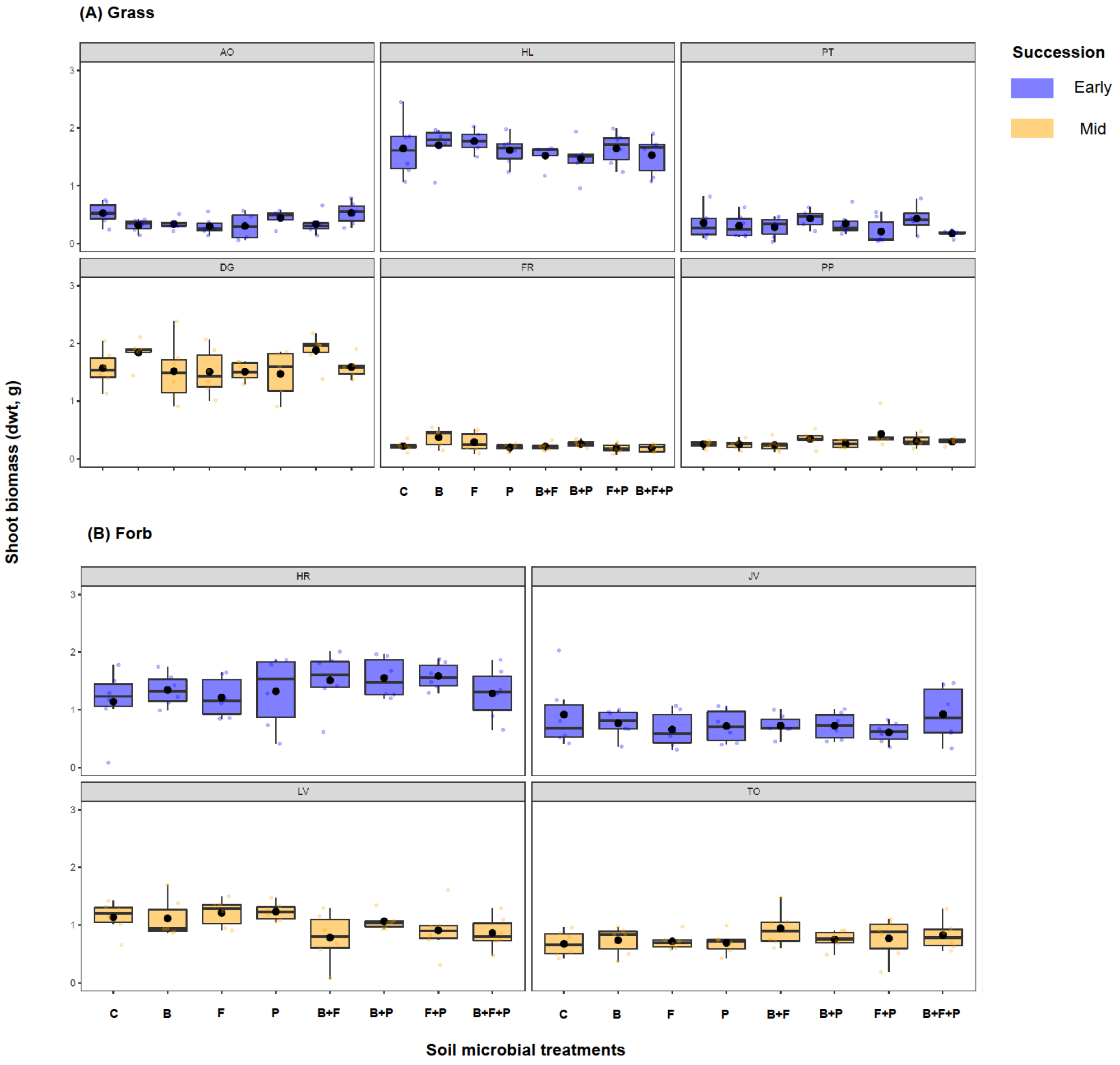

Fig. S3 Shoot biomasses of individual grass species (A) and forb species (B). early- (blue boxplots) and mid-successional (yellow boxplots) are shown. Circles inside boxplots represent the mean, horizontal lines median values. C: Control; B: Bacteria; F: Fungi; P: Protists. Number of observations for each grass species per successional type per microbial treatment = 18; Number of observations for each forb species per successional type per microbial treatment = 12.

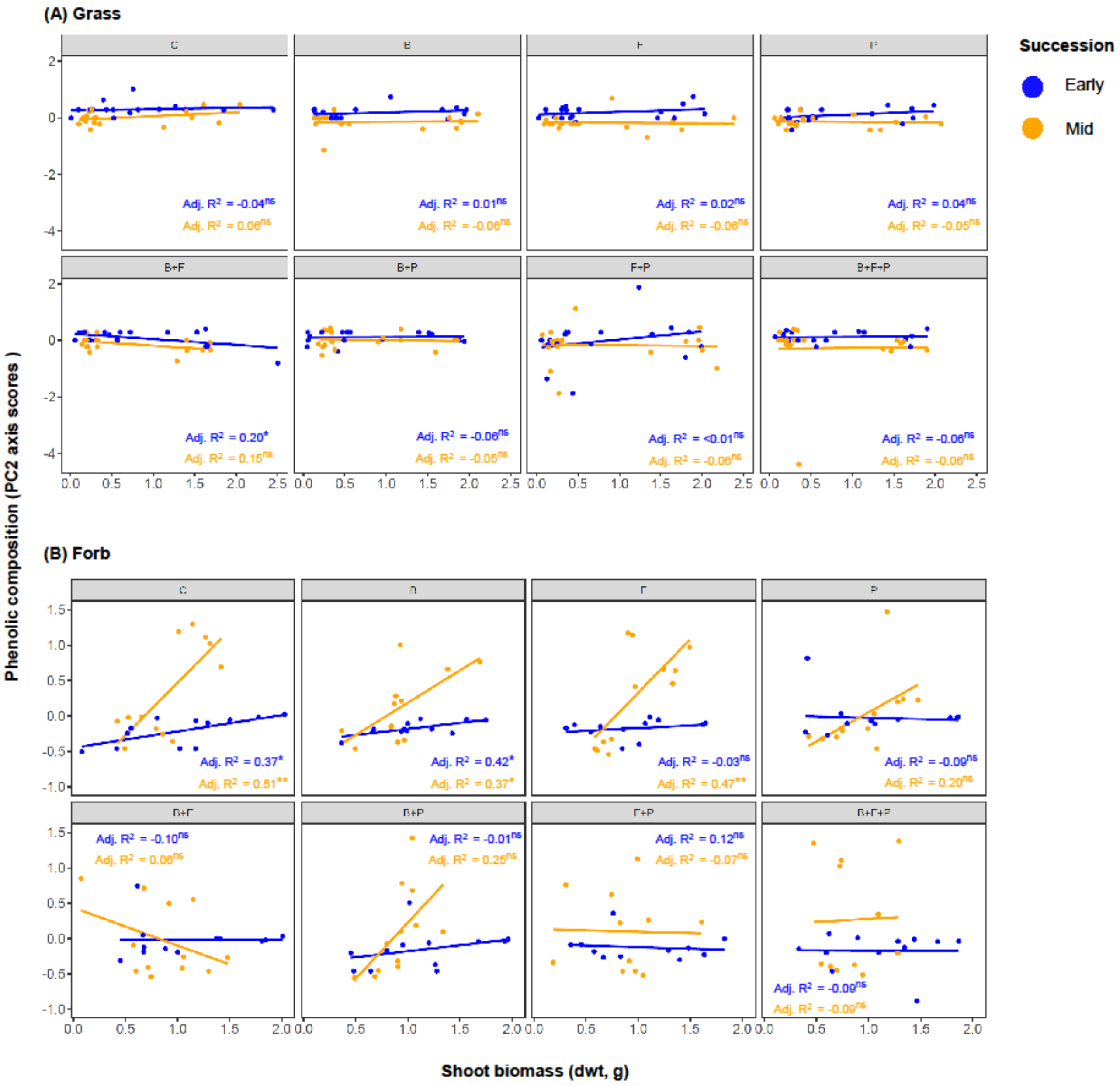

Fig. S4 Composition of phenolics as expressed by PC2 Scores against shoot biomasses (in gram) in grasses (A) and forbs (B). C: Control; B: Bacteria; F: Fungi; P: Protists. Statistically significant correlations are indicated with stars (level of significance with *= $p < 0.05$ and **= $p < 0.01$, while ns representing non-significant differences). Number of observations for grass communities per successional type per microbial treatment = 18; Number of observations for forb communities per successional type per microbial treatment = 12.

Methods S1 Information about fungal and protistan cultivation

To confirm the identity of fungi, DNA was extracted with the Zymo Research Fungal/Bacterial miniprep kit according to the manufacturer's instructions and the entire ITS region was amplified by PCR using primers ITS1 and ITS4 (White et al., 1990) to determine their taxonomic identity. For each fungal culture, we performed PCRs in 25 µL reaction volumes using 1 µL template, 1 µL 25 mM $MgCl_2$, 1 µL 10 µM of each primer, 3,125 2 mM dNTP, 2.5 µl 10xBuffer with $MgCl_2$ and 0,125 µL 5 U [µL]-1 FastStart Taq DNA polymerase (Roche Diagnostics GmbH, Mannheim, Germany) on a thermal cycler equipped with a heated lid. An initial denaturation and enzyme activation step of 10 min at 95°C was followed by amplification for 35 cycles (30 s at 95°C, 30 s at 56°C, 60 s at 72°C) followed by a final 5 min extension at 72°C. PCR products were Sanger sequenced at Macrogen (Amsterdam, The Netherlands). Resulting sequences were compared with known species using BLASTn at against the NCBI database (http://www.ncbi.nlm.nih.gov/Blast.cgi) and sequences thereby manually assigned.
For the experimental setup, the fungal cultures were combined as shown in Extended Methods Table S1. The inoculation scheme of all microbial groups is illustrated in Fig. S1
Protists were morphologically identified and grouped into morphogroups. For the experiment we selected those taxa that we recovered both in early and mid-successional fields and combined in three distinct mixes per successional origin (Extended Methods Table 2).

Extended Methods Table S1. Overview of fungal mixes used in the greenhouse experiment. Each number refer to a cultivated strain shown in Extended Fig. 1.

Stage	Mix	*Trichoderma hamatum*	*Mucor moelleri*	*Mucor hiemalis*	*Fusarium culmorum*	*Fusarium oxysporum*	*Clonostachys rosea*	*Trichoderma* sp.	*Penicillium* sp.	*Mortierella* sp.
Early	1	1	49	55b	38	32a	5	29	63	20
	2	40	49	55b	38	32a	5	29	63	20
	3	25	49	55b	38	32a	5	29	63	20
	4	1	65	55b	38	32a	5	29	63	20
	5	40	65	55b	38	32a	5	29	63	20
	6	25	65	55b	38	32a	5	29	63	20
Mid	1	54	26	13	11	23b	44	23a	7	52
	2	36	26	13	11	23b	44	23a	7	52
	3	22	26	13	11	23b	44	23a	7	52
	4	54	67	13	11	23b	44	23a	7	52
	5	36	67	13	11	23b	44	23a	7	52
	6	22	67	13	11	23b	44	23a	7	52

Extended Methods Table 2. Overview of protist mixes used as inocula. A protist mix contained protists from a different taxon/group from different sites. Two distinct Heterolobosea species were isolated from AK and used to assembly mix 2 and 3. WA: Wageningse Eng Veld 3; WE: Wegberm Paardenwei Telefoonweg 2 Paardenwei Telefoonweg 2; RE: Renkumse Heide; AK: Akker Reijerscamp; OUD: Oud Reemst.

Stage	Mix	Glissomonad	*Colpoda* sp.	*Acanthamoeba sp.*	Heterolobosea
Mid	1	OUD	AK	AK	OUD
	2	OUD	AK	AK	AK
	3	OUD	AK	AK	AK
Early	4	WA	RE	WA	WE
	5	WA	RE	WA	RE
	6	WA	RE	WE	RE

References

White TJ, Bruns T, Lee S, Taylor J 1990. *Amplification and direct sequencing of fungal ribosomal RNA genes for phylogenetics*. In Innis MA, Gelfand DH, Sninsky JJ, White TJ Eds, *PCR Protocols: A Guide to Methods and Applications*. New York, NY, USA: New York Academic Press, 315-322
